# Association of CXCR7 and CXCR4 expression with tumor immune phenotypes in tubo-ovarian high-grade serous carcinoma

**DOI:** 10.1007/s11845-026-04437-5

**Published:** 2026-05-13

**Authors:** Burak Tatar, Sultan Caliskan, Fatma Nur Uygun, Sercan Ergün, Neslihan Hekim, Mehmet Kefeli, Sezgin Gunes

**Affiliations:** 1 Department of Molecular Medicine, Ondokuz Mayis University, Samsun, Türkiye; 2 Department of Pathology, Ondokuz Mayis University, Samsun, Türkiye; 3 Department of Medical Biology, Faculty of Medicine, Ondokuz Mayis University, Samsun, Türkiye; 4 Stem Cell Application and Research Center, Samsun, Türkiye

**Keywords:** Tubo-ovarian high-grade serous carcinoma, CXCR7, CXCL12, CXCR4, Tumor-infiltrating lymphocytes, Immune exclusion

## Abstract

**Background:**

Tumor-infiltrating lymphocytes (TILs) are associated with favorable outcomes in high-grade serous ovarian carcinoma (HGSC); however, the tumor microenvironment may modulate immune cell infiltration. This study aimed to evaluate the association between tumor and stromal expression of CXCL12, tumor expression of CXCR4 and CXCR7, immune phenotypes, and survival outcomes in tubo-ovarian HGSC.

**Methods:**

Eighty-six patients with primary tubo-ovarian HGSC who underwent cytoreductive surgery were retrospectively analyzed. Immunohistochemical expression of CXCL12 (tumor and stroma), CXCR4, and CXCR7 was assessed. TILs were semi-quantitatively scored and dichotomized as low (score 1) or high (scores 2–3). Immune phenotypes were classified as immunogenic, immune-excluded, or immune-ignorant. Recurrence-free survival (RFS) and overall survival (OS) were analyzed using Kaplan–Meier methods, log-rank tests, and Cox regression. Multivariable logistic regression was used to identify factors associated with high TILs.

**Results:**

Median RFS was 23.2 months (95% CI, 19.2–27.1) and median OS was 47.3 months (95% CI, 36.3–55.0). RFS differed significantly across immune phenotypes (log-rank p=0.047), with shorter RFS observed in the immune-excluded phenotype compared with the immunogenic phenotype (hazard ratio, 2.20; 95% CI, 1.14–4.25). High tumor CXCR7 expression was independently associated with a lower likelihood of high TILs (adjusted odds ratio, 0.22; 95% CI, 0.07–0.65). CXCL12 expression was not associated with survival outcomes.

**Conclusion:**

Tumor CXCR7 expression is associated with reduced lymphocytic infiltration and an immune-excluded phenotype in HGSC, suggesting a potential role of the CXCR7 axis in shaping the tumor immune microenvironment.

## Introduction

Despite advancements in immunotherapy, significant impact has not been achieved in tubo-ovarian high-grade serous carcinoma (HGSC) due to the suppressive nature of the tumor microenvironment. Unlike tumors with a high mutation burden like melanoma and lung cancer, HGSC exhibits limited responses to immune checkpoint inhibitors. HGSC can be categorized into activated and inhibited immune-response groups, with tumor-infiltrating lymphocytes (TILs) absent in 45% and present in 55% of cases [1]. Notably, TILs positivity is associated with improved survival outcomes [1].

The chemokine family, including the C-X-C chemokine receptor type 4 receptor (CXCR4) for C-X-C motif chemokine 12 ligand (CXCL12), additionally known as stromal cell-derived factor-1 (SDF-1), plays a key role in regulating cell traffic between tissues. Increased CXCL12 levels in damaged tissues attract circulating endothelial progenitor cells to facilitate tissue repair. Because cancer is often described as a “non-healing wound,” the CXCL12–CXCR4 axis has garnered attention for its role in promoting cancer cell migration, metastasis, and immunosuppression within the tumor microenvironment [2, 3]. CXCR4 is overexpressed in more than 23 human malignancies, including breast cancer, ovarian cancer, melanoma, and prostate cancer [4]. Although normal ovarian tissue typically exhibits low or negligible CXCR4 expression [5, 6], metastatic HGSC frequently shows higher levels of CXCR4 [7, 8]. A recent report suggests that elevated CXCL12 is associated with reduced progression-free survival [9]. In addition, high intratumoral CXCL12 enhances the tumor-promoting function of immunosuppressive CXCR4 + regulatory T cells (Tregs) and plasmacytoid dendritic cells (pDCs) [3, 10]. Dual blockade of the CXCL12–CXCR4 axis and the PD-1/PD-L1 checkpoint has shown more effective tumor growth control and improved survival than either treatment alone [11].

Although CXCR4 was initially considered the sole receptor for CXCL12, mounting evidence indicates that CXCR7 can also bind CXCL12, modulating angiogenesis and tumor progression [12]. The prognostic value of CXCR7 remains controversial, with some studies linking its expression to worse outcomes and others reporting the opposite [13].

In this study, we classified HGSC by immunogenicity (based on TILs and CD8 staining) and evaluated the functional role of the CXCR4–CXCR7–CXCL12 pathway. We assessed the prognostic significance of CXCL12 expression and examined how CXCR4 and CXCR7 expression in the tumor microenvironment might influence CXCL12 levels. We also hypothesized that the CXCL12–CXCR4–CXCR7 chemokine axis modulates immune cell in HGSC. This study aimed to characterize expression patterns of these receptors in relation to TIL density and evaluate their prognostic significance.

## Materials and methods

### Study population

This retrospective study was conducted in the Departments of Medical Biology and Medical Pathology at Ondokuz Mayis University. Sample-size calculations were performed in Minitab based on results from a similar study [1]. A minimum of 86 participants (43 per group) was required to detect a significant difference with a 5% Type I error and 80% power. The cohort comprised patients diagnosed with tubo-ovarian HGSC within the past 10 years at the same institution. Data were obtained from formalin-fixed, paraffin-embedded (FFPE) tumor samples and patient clinical records, including demographics, tumor stage and grade, recurrence-free survival (RFS), and overall survival.

Inclusion criteria included:

- Histopathologically confirmed tubo-ovarian HGSC.
- Macroscopic intra-abdominal/peritoneal metastasis > 2 cm (Stage IIIC or IV).
- Treatment with either neoadjuvant chemotherapy followed by interval cytoreductive surgery or primary cytoreductive surgery followed by adjuvant chemotherapy (paclitaxel–carboplatin).
- Chemotherapy resistance was defined as disease progression during platinum-based chemotherapy or recurrence within 6 months after completion of platinum-based chemotherapy.
- Residual tumor size < 1 cm after cytoreductive surgery.

Exclusion criteria included:

- Non-standard chemotherapy regimens (i.e., non-platinum-based).
- Suboptimal cytoreduction with macroscopic residual disease at upfront surgery.
- Death unrelated to the disease (e.g., accidents, natural disasters).

### Immunohistochemistry

The FFPE sections were analyzed using immunohistochemistry (IHC) to detect protein expression. A mouse monoclonal antibody against CD8 [C8/144B] (Biocare, CA, USA) was used to identify the 32 kDa CD8 antigen. This method enabled quantification of CD8^+^ TILs, which provide a key indicator of tumor immune response. All histopathological and immunohistochemical slides were independently reviewed by two pathologists. Because of the retrospective design and the use of archival material linked to clinicopathologic records, scoring was not performed blinded to clinical outcomes.

TILs were semi-quantitatively evaluated in hematoxylin–eosin–stained sections and graded according to the number of T lymphocytes per high-power field (HPF) as follows: score 1+, < 6 T cells per HPF; score 2+, 6–19 T cells per HPF; and score 3+, ≥ 20 T cells per HPF. For statistical analyses, TIL scores were dichotomized as low (score 1+) and high (scores 2 + and 3+).

Based on combined CD8 + T-cell density and TIL scores, tumors were further classified into three immune phenotypes: “immunogenic”, defined as high CD8 + T-cell infiltration (IHC 3+); “immune-excluded”, defined as medium or low CD8 + T-cell density (IHC 1 + or 2+) in the presence of medium or high TIL levels (IHC 2 + or 3+); and “immune-ignorant”, defined as medium or low CD8 + T-cell density (IHC 1 + or 2+) with low TIL levels (IHC 1+).

Chemokine receptors: Rabbit polyclonal antibodies against SDF1/CXCL12, CXCR4, and CXCR7 (Medaysis, CA, USA) were used to detect these markers. Staining was evaluated using two parameters: staining intensity and extent/distribution of positive staining. For both parameters, scores were assigned as 0 (negative), 1 (weak/focal), or 3 (strong/diffuse). For SDF1/CXCL12, stromal and metastatic tumor tissues were evaluated separately; thus, the extent score referred to positive stromal cells in stromal areas and positive tumor cells in metastatic tumor areas, respectively. CXCR4 and CXCR7 were assessed only in metastatic tumor sections, and the extent score referred to positive tumor cells. A combined score (range: 0–6) was derived by summing the intensity and extent scores. Samples with a total score of 0–3 were classified as low expression, whereas those with scores of 4–6 were categorized as high expression.

#### CXCL12 expression evaluation

RNA was extracted from FFPE tumor sections (5 μm thick) using the EZ-10 Spin Column Total RNA Mini-Preps Kit (Biobasic, Canada). After deparaffinization in xylene, the sections were rehydrated, lysed in RLT buffer, and purified on a spin column with sequential wash steps. The eluted RNA was reverse-transcribed into cDNA using a protocol that included initial primer annealing at 25 °C, reverse transcription at 55 °C, and enzyme inactivation at 85 °C.

Quantitative PCR (qPCR) was performed using a 2×Real-Time PCR Master Mix on a Rotor-Gene Q platform (Qiagen, Germany). Relative expression levels of CXCL12 were calculated with the 2^−ΔΔCt^ method, using GAPDH as the internal control [14].

### Statistical analysis

All statistical analyses were performed with SPSS version 21.0 (IBM, Armonk, NY, USA). Pearson correlation tests were used to evaluate associations between CXCL12 expression levels and chemokine receptor positivity. Kaplan–Meier survival estimates, and Cox proportional hazards models were applied to assess RFS and overall survival (OS), with the log-rank test used to compare survival curves. Multivariate analyses were adjusted for relevant covariates, reporting hazard ratios (HRs) and 95% confidence intervals (CIs). Logistic regression was employed to examine the relationship between immune profiles and receptor expression. Statistical significance was defined as *p* < 0.05.

This study was conducted and reported in accordance with the STROBE guidelines for observational studies.

## Results

### Patient demographics and clinical characteristics

A total of 86 patients met the inclusion criteria. The mean age was 57 years (range, 35–84; SD, ± 10.8). The median CA125 value was 1020 (range, 109–9311; SD, ± 1749). Sixty-three patients (73%) underwent initial (primary) surgery and were subsequently given chemotherapy. Neoadjuvant chemotherapy was administered to 23 patients (27%), who subsequently underwent surgery. All patients received paclitaxel and carboplatin chemotherapy, either in the adjuvant or neoadjuvant setting. Eighteen patients (21%) met the definition of chemotherapy-resistant disease, defined as progression during platinum-based chemotherapy or recurrence within 6 months after completion of treatment, and subsequently received second-line therapy.

The median RFS was 23.2 months (95% CI: 19.2–27.1 months), and the median overall survival was 47.3 months (95% CI: 36.3–55.0 months). Twenty-two patients were alive at the end of the study, and the rest of the patients died before the study ended. Tumor recurrence within 60 months occurred in 72 patients; in 13 patients, tumor recurrence occurred beyond 60 months.

**Fig. 1:**
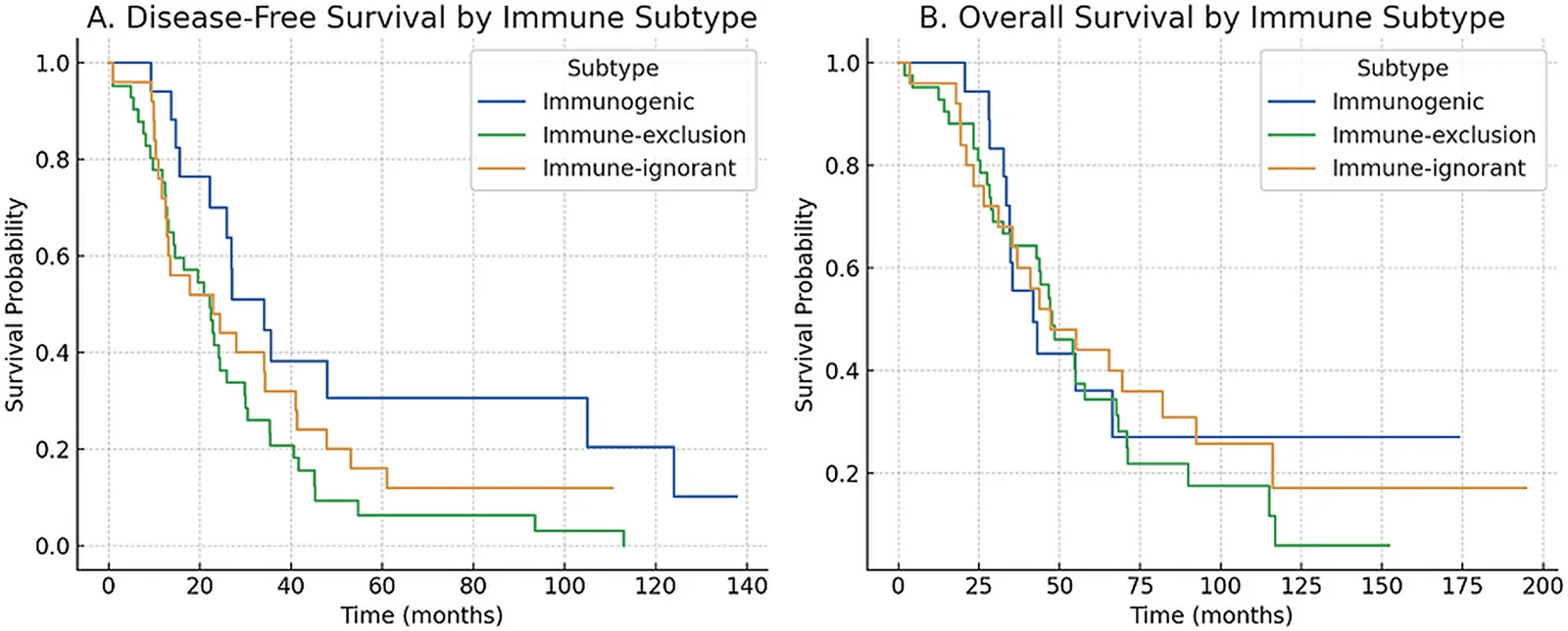
Kaplan–Meier survival analysis stratified by tumor immune subtype (immunogenic, immune-excluded, immune-ignorant). (**A**) Disease-free (recurrence-free) survival curves. (log-rank *p* = 0.047) (**B**) Overall survival curves showing no significant differences

Recurrence-free survival was significantly longer in immunogenic tumors (high CD8^+^ density) compared to non-immunogenic tumors (immune-excluded and immune-ignorant phenotypes with moderate/low CD8^+^ density) (*p* < 0.05). The median RFS was 34 months for the immunogenic group, 22.3 months for the immune-excluded group, and 23 months for the immune-ignorant group (Fig. 1). Figure 1 shows that RFS was longer in the immunogenic group, whereas OS did not differ significantly among the three immune phenotypes. CD8 + lymphocyte-rich tumors were more likely to recur after 60 months (*p* < 0.01; binary logistic regression, odds ratio [OR]: 17.12; 95% confidence interval [CI]: 2.01–141.1).

### Immunohistochemical and real-time PCR results

**Fig. 2:**
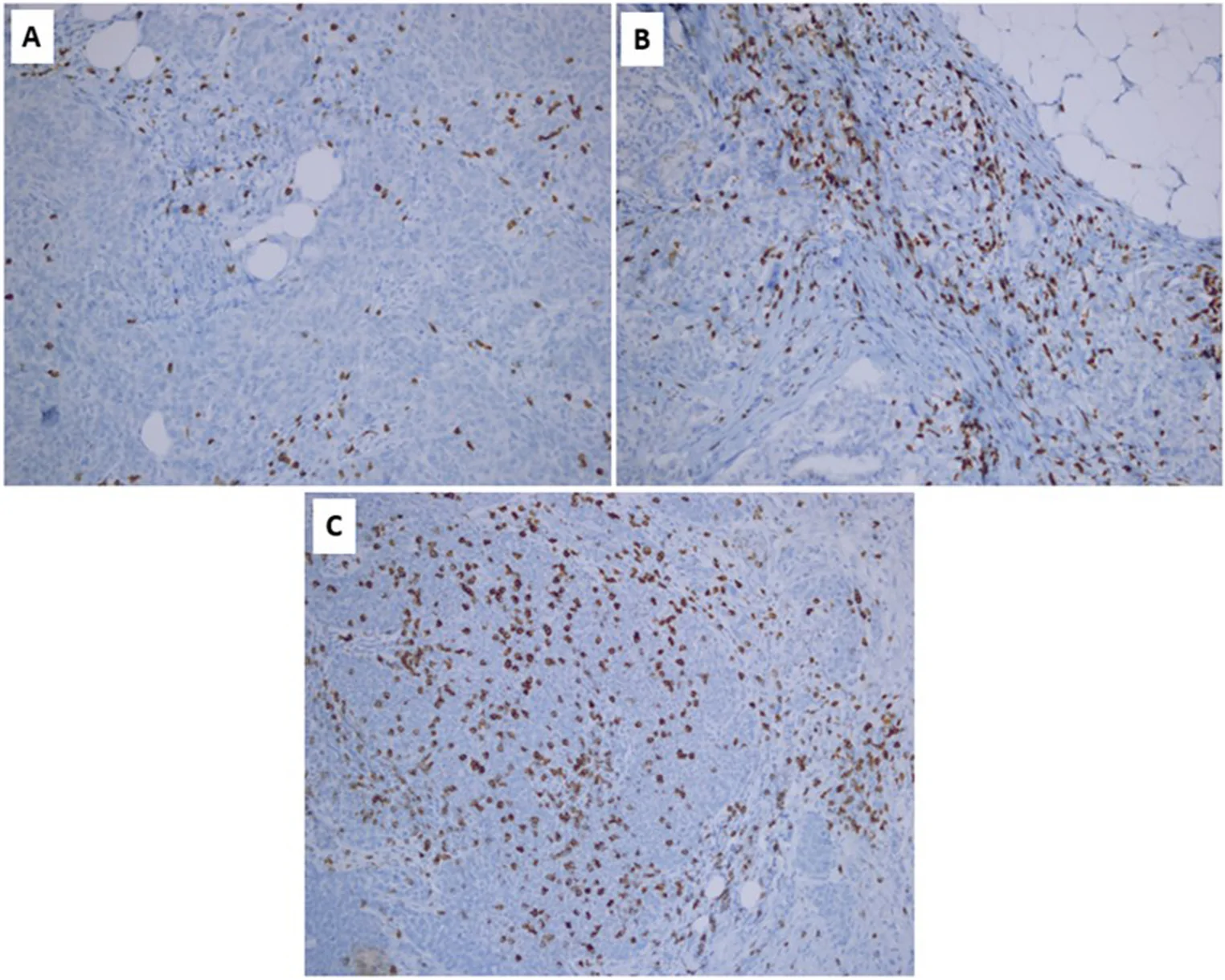
Representative CD8 immunohistochemical staining patterns used for TIL scoring in tubo-ovarian high-grade serous carcinoma. (**A**) Staining in 1–10% of tumor-infiltrating lymphocytes (TILs) (score: 1), (**B**) Staining in 10–50% of TILs (score: 2), (**C**) Staining in more than 50% of TILs (score: 3)

**Fig. 3:**
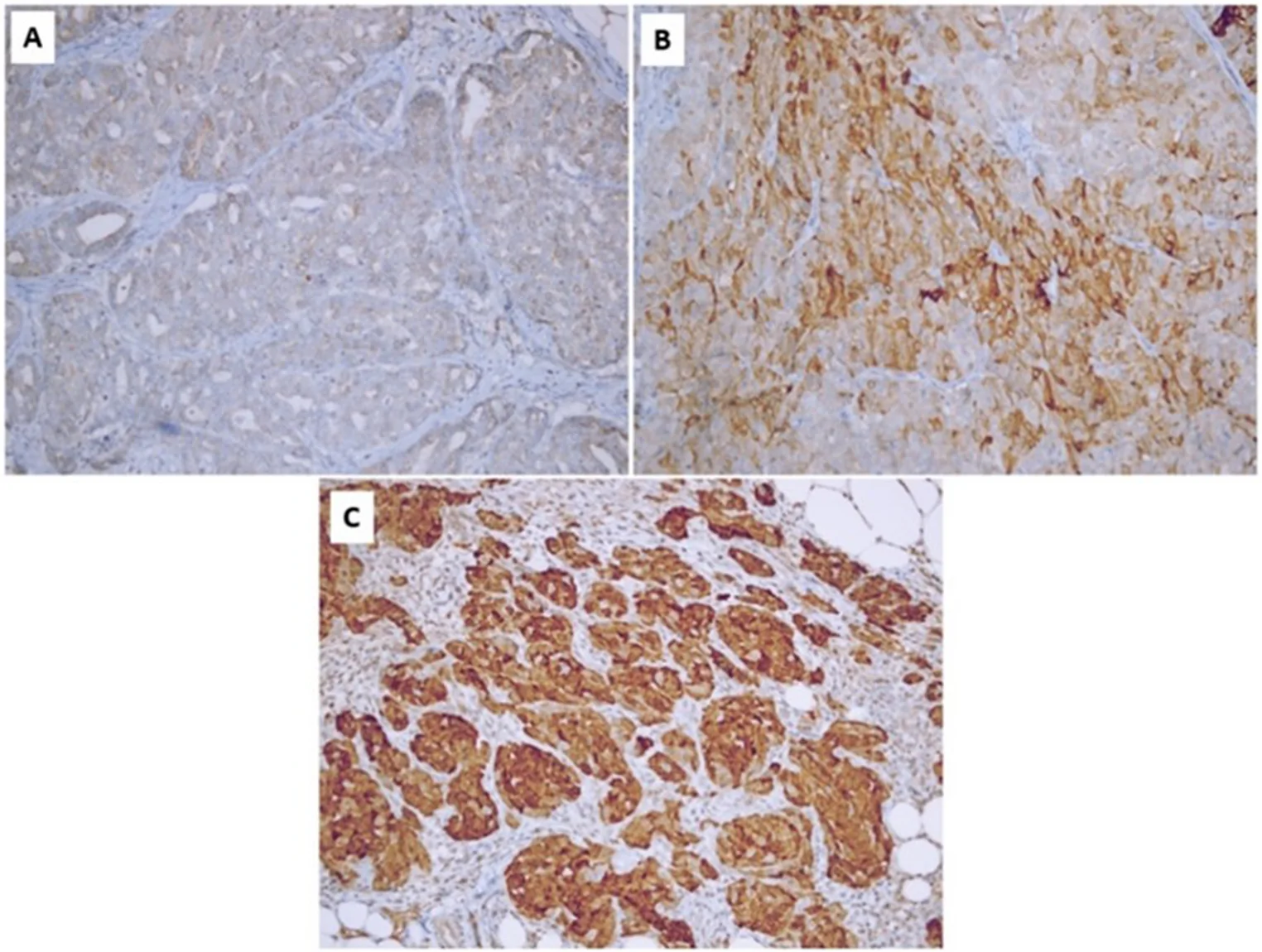
Representative immunohistochemical staining patterns for CXCL12 in metastatic tumor tissue. (**A**) Focal weak staining (score: 1), (**B**) Diffuse weak staining (score: 2), (**C**) Diffuse strong staining (score: 3)

Representative CD8 immunohistochemical staining patterns used for TIL scoring are shown in Fig. 2, illustrating the staining categories applied for TIL scoring. Representative immunohistochemical staining patterns for CXCL12 are shown in Fig. 3. CXCL12, CXCR4, and CXCR7 were all assessed immunohistochemically using the same semiquantitative approach based on focal/weak and diffuse/strong staining patterns. A positive correlation was observed between TIL density and CXCL12 expression (IHC) in the stroma. In contrast, TIL density was negatively correlated with CXCR7 expression, indicating that high CXCR7 levels are inversely associated with TIL presence (*p* < 0.01; OR: 0.17; 95% CI: 0.05–0.56)(Table 1).

**Table 1:** Results of multivariate binary logistic regression evaluating the association betweenchemokine axis components and high intratumoral TIL density (IHC score ≥ 3+) in tubo-ovarian high-grade serouscarcinoma (n=86)

Independent Variables	*P*-value	Odds Ratio (OR)	95% Confidence Interval (CI)
Tumor CXCR7	0.004	0.17	0.05–0.56
Stromal CXCL12	0.375	1.62	0.56–4.75
Tumor CXCL12	0.121	5.37	0.64–44.82
Tumor CXCR4	0.141	0.18	0.02–1.77

Odds ratios (OR) with 95% confidence intervals (CI) and p-values are shown. Tumor CXCR7 high expression was significantly associated with lower odds of TIL infiltration (OR 0.17, 95% CI 0.05–0.56, *p* = 0.004). Abbreviations: *CXCL12* stromal cell-derived factor-1, *CXCR4/7* C-X-C chemokine receptors 4 and 7, *IHC* immunohistochemistry, *TIL* tumor-infiltrating lymphocyte

**Fig. 4:**
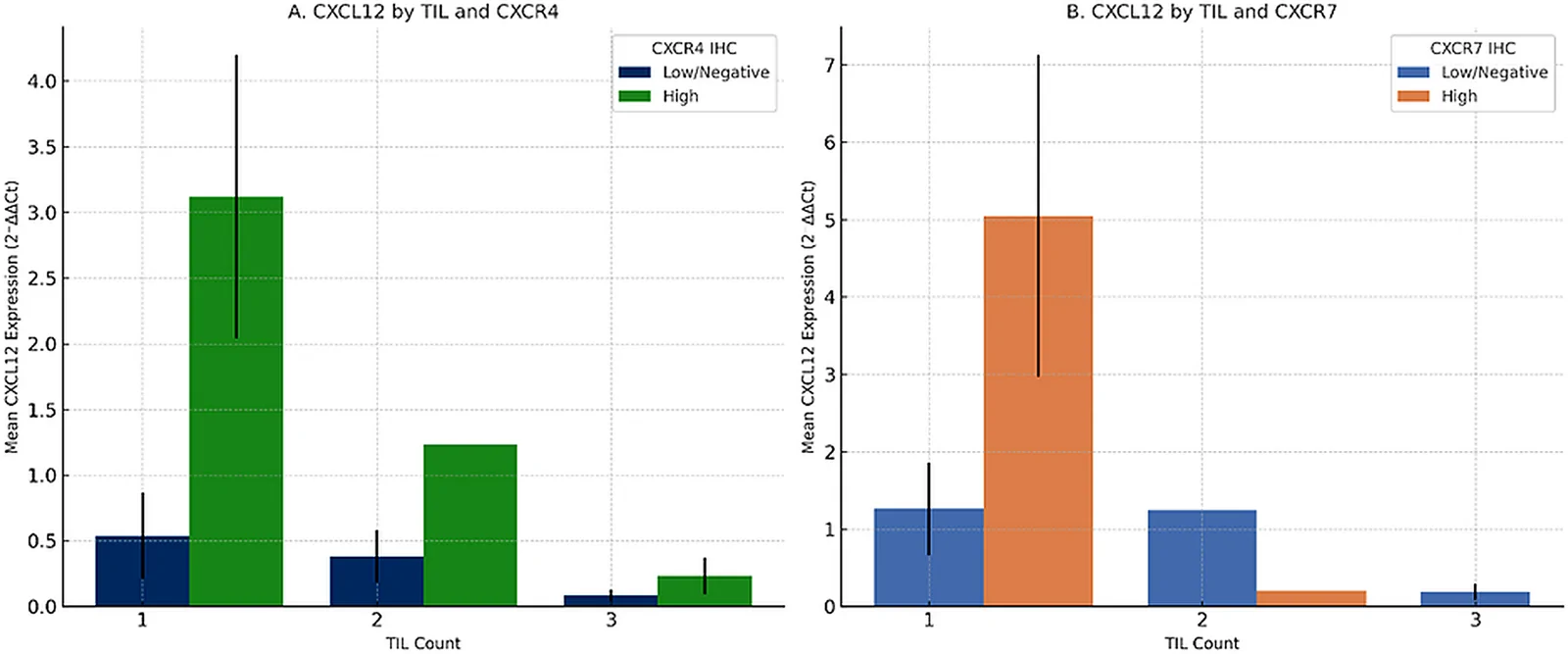
CXCL12 mRNA expression according to TIL density and chemokine receptor expression. (**A**) Mean CXCL12 expression (2⁻ΔΔCt) across TIL categories, stratified by CXCR4 immunohistochemical staining (Low/Negative in dark blue vs. High in green), (**B**) Mean CXCL12 expression across TIL count groups, stratified by CXCR7 expression status (Low/Negative in blue vs. High in orange). Error bars represent the standard error of the mean (SEM)

Multivariate logistic regression showed that neither CXCR4 nor CXCR7 served as a moderator of the relationship between CXCL12 mRNA (fold-change) and TIL density (*p* > 0.05 for both). Notably, CXCR7 expression (by IHC) independently influenced TIL infiltration (*p* < 0.05) (Fig. 4). Neither CXCR7 nor CXCR4 significantly moderates the relationship between CXCL12 fold change and TIL density (*p* > 0.05, multivariate logistic regression; OR: 2.1 [0.29–15.5], OR: 0.43 [0.057–3.2], respectively). However, high CXCR7 expression independently reduces TIL infiltration (*p* < 0.01, binary logistic regression; OR: 0.17 [0.05–0.56]).

**Fig. 5:**
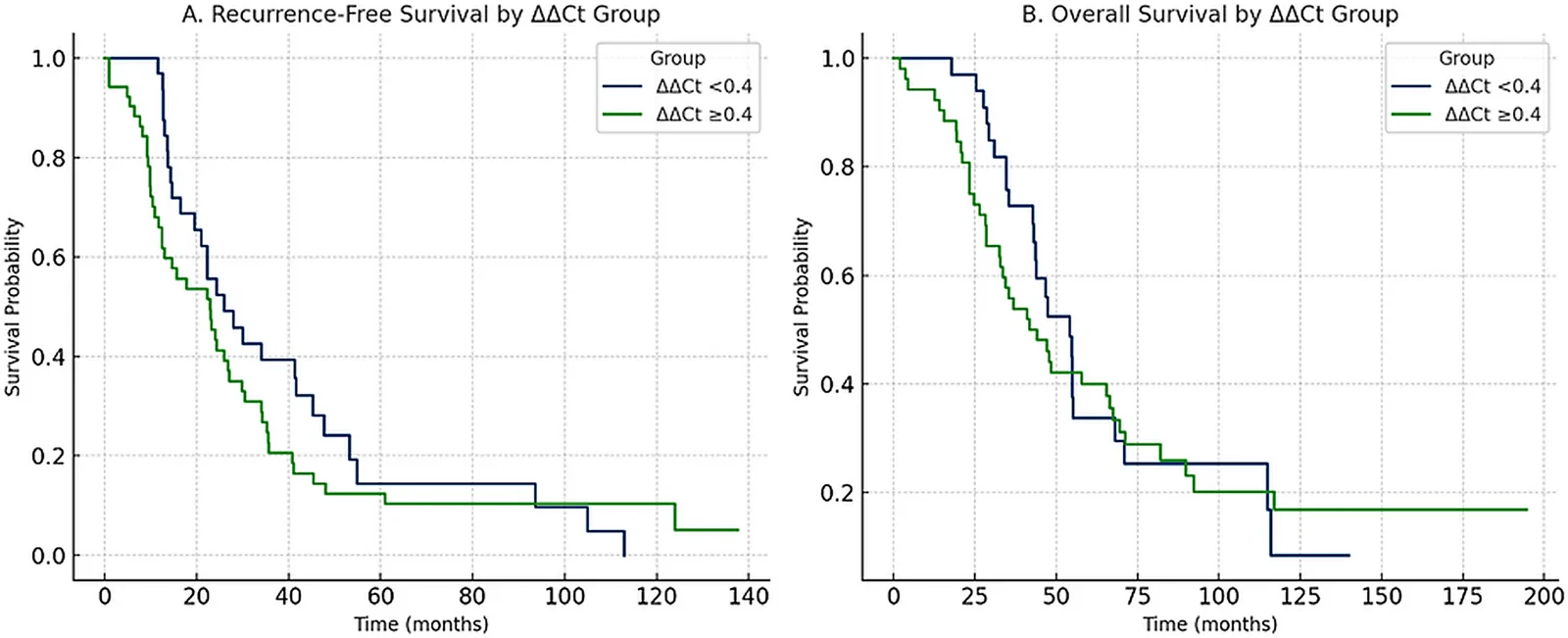
Kaplan–Meier survival analysis stratified by CXCL12 expression (ΔΔCt) (threshold 0.4). (**A**) Recurrence-free survival curves for CXCL12 ΔΔCt < 0.4 (dark blue) vs. CXCL12 ΔΔCt ≥ 0.4 (green) groups. (**B**) Overall survival curves for the same ΔΔCt stratification

Neoadjuvant chemotherapy did not affect CXCL12 expression (binary logistic regression, OR: 1.37; 95% CI: 0.35–5.34). Regarding CXCL12 mRNA levels, 12% of patients showed a very high fold change (> 5-fold), whereas 29% exhibited a very low fold change (< 0.1-fold). Although no significant difference in CXCL12 fold-change values was found among immunogenic, immune-excluded and immune-ignorant tumors (Kruskal–Wallis test, *p* = 0.078), a fold change ≥ 5 for CXCL12 was associated with higher TIL density (*p* < 0.05, Pearson correlation). Kaplan–Meier analysis revealed no significant differences in RFS or OS between patients with high (≥ 0.4-fold) versus low (< 0.4-fold) *CXCL12* mRNA expression (log-rank test, *p* = 0.25 for RFS and *p* = 0.65 for OS) (Fig. 5).

## Discussion

The tumor microenvironment, particularly TILs, plays a critical role in the biology and treatment response of serous carcinomas. In this study, tumors enriched in cytotoxic CD8 + lymphocytes showed prolonged RFS, and high CD8^+^ density was associated with recurrence beyond 60 months. These findings align with prior reports that link TILs, especially CD8 + T cells, to favorable survival outcomes and robust tumor-specific immune responses [15–17].

Conflicting data exist regarding the prognostic significance of CXCL12 [18, 19]. In our cohort, 12% of patients had high CXCL12 expression (≥ 5-fold), which aligns reasonably well with a prior study reporting a 20% rate of high CXCL12 expression [18]. Here, CXCL12 alone did not predict survival; however, levels exceeding a certain threshold (fold change ≥ 5) correlated positively with TIL density—albeit without a similar rise in CD8 + lymphocytes. Moreover, no relationship was found between CXCR4 expression and RFS or overall survival in our cohort. This finding is consistent with other current studies in the literature [9]. In general, *CXCL12* expression in tumors acts as a chemoattractant for T cells via CXCR4 [4, 20]. Indeed, mouse models of melanoma and ovarian cancer have established that CXCL12 can exert dose-dependent immune effects: low levels bolster T-cell infiltration, slow tumor progression, and foster tumor-specific CD8^+^ T-cell responses, whereas high levels diminish antigen-specific T-cell infiltration and promote immunosuppression through Treg accumulation [20].

Our data showed a positive correlation between TIL density and CXCL12 expression above a certain threshold (fold change ≥ 5). This increase was not accompanied by a corresponding rise in CD8 + cells. These findings suggest that high CXCL12 levels may preferentially recruit immunosuppressive Tregs and thereby promote an immunosuppressive tumor microenvironment in epithelial ovarian cancer. This concept is further supported by an orthotopic murine ovarian cancer model, where experimentally reduced CXCL12 levels led to decreases in myeloid cells, plasmacytoid dendritic cells, and Tregs, accompanied by increased infiltration of tumor-specific T lymphocytes [21].

Additionally, tumor CXCR7 expression was associated with a lower total TIL count (*P* < 0.01), whereas CXCR4 expression correlated with lower CD8 + lymphocyte counts (*P* < 0.05). These findings suggest that the availability of free CXCL12 for CXCR4 receptors on T lymphocytes may be reduced in the tumor microenvironment.

The apparent discrepancy between the non-significant association of CXCR4 with overall TIL density and its significant inverse association with CD8 + density likely reflects endpoint heterogeneity rather than a true contradiction. Overall TILs aggregate multiple lymphocyte subsets, whereas CD8 + cells capture the effector compartment most directly linked to antitumor activity; thus, CXCR4 may preferentially affect effector composition, while CXCR7 may impact global lymphocyte entry.

In a pancreatic cancer model, the drug AMD3100, which blocks the interaction between CXCR4-positive tumor cells and CXCL12-producing fibroblasts, led to a quick increase in T-cell accumulation [22]. Although CXCR7 is not expressed in human and mouse leukocytes and thus does not directly inhibit T cell infiltration, it significantly influences tumor proliferation, angiogenesis, and migration [13, 23, 24]. Evidence suggests that CXCR7 may function as a “decoy” receptor lacking classical Gi signaling activity [25] and effectively sequesters CXCL12. Thereby, it modulates its interaction with CXCR4. Notably, our data indicate that even with high CXCL12 levels, substantial CXCR7 expression (as detected by IHC) correlates with fewer TILs and lower CD8^+^ lymphocyte counts (*p* < 0.05), supporting the concept of CXCR7 as a decoy receptor that restrains lymphocyte infiltration into the tumor.

Although we observed a positive correlation between CXCL12 expression exceeding a fivefold increase and TIL infiltration, this relationship is unlikely to be strictly linear. It can be hypothesized that CXCL12 levels above a certain threshold circumvent receptor-mediated sequestration, making it more freely available to enhance TILs recruitment. Supporting this notion, a recent pancreatic cancer study found that exogenous CXCL12 administration boosted CD8 + T-cell numbers and re-established an immunogenic tumor response [26]. Other investigations have shown indirectly that CXCL12 fosters immune escape in pancreatic cancer, based on AMD3100-mediated CXCR4 blockade [22, 27]. Additionally, CXCR4 blockade increased circulating CD8 + T cells in a mouse sepsis model. In hepatocellular carcinoma models, it reduced immunosuppression, tumor growth, and metastasis; however, the number of CD8 + TILs remained unchanged [28].

Our results indicate that neoadjuvant chemotherapy does not alter CXCL12 expression, which is consistent with a previous report [29]. However, neoadjuvant chemotherapy is generally administered to patients with initially unresectable tumors. Therefore, an important limitation of our study is the inability to compare paired pre- and post-treatment tissues from the same patient. Despite the absence of significant changes in CXCL12 levels, patients who received neoadjuvant therapy exhibited higher CD8^+^ T-cell infiltration.

Finally, the retrospective design, lack of uniform criteria for administering chemotherapy, and reliance on archival tissues are notable limitations. By focusing on specific components of the chemokine axis (CXCR4, CXCR7, and CXCL12), we may be overlooking additional immune determinants such as other chemokines and immune checkpoints like PD-1/PDL-1.

## Conclusion

In HGSC, CXCL12 expression was not an independent prognostic factor. High tumor CXCR7 expression was associated with reduced TIL infiltration, while CXCR4 expression was inversely associated with CD8 + lymphocyte density. These findings support a role for the CXCR7/CXCR4 axis in shaping the tumor immune microenvironment and suggest that CXCR7 may represent a potential therapeutic target for future investigation.

## Data Availability

De-identified data are available from the corresponding author upon reasonable request, subject to institutional and ethical approvals.
